# Tackling functional redundancy of Arabidopsis fatty acid elongase complexes

**DOI:** 10.3389/fpls.2023.1107333

**Published:** 2023-01-25

**Authors:** Marguerite Batsale, Marie Alonso, Stéphanie Pascal, Didier Thoraval, Richard P. Haslam, Frédéric Beaudoin, Frédéric Domergue, Jérôme Joubès

**Affiliations:** ^1^ Univesity of Bordeaux, CNRS, LBM, UMR 5200, Villenave d’Ornon, France; ^2^ University of Bordeaux, INRAE, BFP, UMR 1332, Villenave d’Ornon, France; ^3^ Plant Sciences, Rothamsted Research, Harpenden, United Kingdom

**Keywords:** yeast, *Arabidopsis thaliana*, *Nicotiana benthamiana*, VLCFA(very-long-chain fatty acids), KCS gene family

## Abstract

Very-long-chain fatty acids (VLCFA) are precursors for various lipids playing important physiological and structural roles in plants. Throughout plant tissues, VLCFA are present in multiple lipid classes essential for membrane homeostasis, and also stored in triacylglycerols. VLCFA and their derivatives are also highly abundant in lipid barriers, such as cuticular waxes in aerial epidermal cells and suberin monomers in roots. VLCFA are produced by the fatty acid elongase (FAE), which is an integral endoplasmic reticulum membrane multi-enzymatic complex consisting of four core enzymes. The 3-ketoacyl-CoA synthase (KCS) catalyzes the first reaction of the elongation and determines the chain-length substrate specificity of each elongation cycle, whereas the other three enzymes have broad substrate specificities and are shared by all FAE complexes. Consistent with the co-existence of multiple FAE complexes, performing sequential and/or parallel reactions to produce the broad chain-length-range of VLCFA found in plants, twenty-one KCS genes have been identified in the genome of *Arabidopsis thaliana*. Using CRISPR-Cas9 technology, we established an expression platform to reconstitute the different Arabidopsis FAE complexes in yeast. The VLCFA produced in these yeast strains were analyzed in detail to characterize the substrate specificity of all KCS candidates. Additionally, Arabidopsis candidate proteins were transiently expressed in *Nicotiana benthamiana* leaves to explore their activity and localization *in planta*. This work sheds light on the genetic and biochemical redundancy of fatty acid elongation in plants.

## Introduction

Very-long chain fatty acids (VLCFA) are defined as fatty acids with a carbon chain with more than 18 carbon atoms. In plants, they are found with a wide range of chain-lengths (up to 38 carbon atoms), and with various degrees of unsaturation or hydroxylation levels (Batsale et al., 2021; Kyselová et al, 2022). VLCFA are required in all plant cells for the biosynthesis of membrane lipids such as phospholipids, especially phosphatidyl-serine, which is enriched in saturated C20, C22 and C24 VLCFA, but also phosphatidyl-choline and -ethanolamine (Li-Beisson et al., 2013). Sphingolipids, which specifically accumulate in the outer leaflet of the plasma membrane, contain α-hydroxylated saturated and monounsaturated C24 and C26 VLCFA. VLCFA can also be incorporated in seed storage triacylglycerols (TAGs), and represent up to 27% of the total acyl-chains in Arabidopsis seeds (Batsale et al., 2021). VLCFA are also precursors for the synthesis of lipid barriers such as cuticular waxes and suberin in aerial organs epidermis and roots, respectively. Although variable amounts of free VLCFA are generally quantified in the cuticular waxes between plant species, the wide variety of aliphatic wax compounds is uniquely derived from VLCFA with chain-length ranging from 24 to 38 carbon atoms (Bernard and Joubès, 2013). Most wax VLCFA derivatives are saturated, however minor amounts of unsaturated alcohols and alkenes, as well as branched molecules can also be found (Busta and Jetter, 2017). Root suberin contains high amounts of VLCFA and VLCFA-derived ω-hydroxylated fatty acids, α,ω-dicarboxylic acids, alcohols and alkyl-hydroxycinnamates (Franke and Schreiber, 2007; Delude et al., 2016). Plant VLCFA are therefore at the origin of a high diversity of lipids found in various subcellular and tissular locations. As such, VLCFAs are critical for multiple physiological processes such as cell division and differentiation, intracellular trafficking, cuticular conductance or energy storage, and play several roles in plant stress response (Zhukov and Shumskaya, 2020; Batsale et al., 2021).

VLCFA result from the endoplasmic reticulum (ER)-associated acyl-CoA elongase activity carried out by the multi-enzymatic fatty acid elongase (FAE) complexes. Each elongation cycle consists of four successive reactions generating an acyl-chain extended by two carbons (Haslam and Kunst, 2013). The first step, catalysed by the 3-ketoacyl-CoA synthase (KCS), consists in condensing an acyl-CoA with a malonyl-CoA. The resulting 3-ketoacyl-CoA is then reduced by a 3-ketoacyl-CoA reductase (KCR) into 3-hydroxyacyl-CoA, which is dehydrated by a 3-hydroxacyl-CoA dehydratase (HCD) to form a trans-2,3-enoyl-CoA. The trans-2,3-enoyl-CoA is then reduced by the trans-2,3-enoyl-CoA reductase (ECR) leading to the formation of an acyl-CoA elongated by two carbons.

Studies, including photoperiod and chemical inhibition of elongase activities, suggest that multiple elongases with distinct chain-length specificities co-exist producing the broad chain-length-range of VLCFA, the KCS being the key rate-limiting enzyme (Von Wettstein-Knowles, 1982). Indeed, the KCR, HCD and ECR enzymes have a broad substrate specificity and are shared by all FAE complexes (Kunst and Samuels, 2009). Yeast mutant complementation assays showed that Arabidopsis KCR1 and PASTICCINO2 (PAS2) encode a functional 3-ketoacyl-CoA reductase and 3-hydroxacyl-CoA dehydratase, respectively (Bach et al., 2008; Beaudoin et al., 2009). Complementation by CER10 of the Arabidopsis *cer10* and the yeast *tsc13-1elo2Δ* mutants demonstrated that *CER10* encodes a functional trans-2,3-enoyl-CoA reductase (Zheng et al., 2005). Consistent with the hypothesis that the three enzymes have broad substrate specificities and are shared by all FAE, the inactivation of KCR1 or PAS2 is embryo lethal, whereas the inactivation of CER10 as well as partial loss of KCR1 or PAS2 activity result in a severe reduction of several classes of VLCFA-containing lipids (waxes, triacylglycerols and sphingolipids) causing important developmental defects (Zheng et al., 2005; Bach et al., 2008; Beaudoin et al., 2009).

In contrast to the KCR, HCD and ECR enzymes, each encoded by one or two genes, more than twenty KCS members have been annotated in several angiosperm genomes supporting the idea that multiple FAE complexes co-exist in plant cells: 21 putative KCS isoenzymes were identified in Arabidopsis (Joubès et al., 2008), 25 in sorghum (Zhang et al., 2022), 26 in maize (Campbell et al., 2019), 30 in peanut (Huai et al., 2020), and as many as 58 in cotton and rapeseed (Xiao et al., 2016; Xue et al., 2020).

Although they have been thoroughly studied in Arabidopsis the exact biochemical activity and physiological functions of most KCS remain unknown. Only nine out of the 21 Arabidopsis *kcs* T-DNA mutant lines show a lipid phenotype (Haslam and Kunst, 2013; Hegebarth et al, 2017; Kim and Lee, 2021; Huang et al, 2022) suggesting that overlapping substrate specificities and expression patterns between the different KCS may lead to functional redundancies. Similarly, out of the 18 Arabidopsis KCS that have been heterologously expressed in yeast at this time, only nine were functionally characterized (Paul et al., 2006; Tresch et al., 2012; Hegebarth et al., 2017). It should be pointed out that it has often been the same KCS that showed activity in yeast and an Arabidopsis mutant phenotype, so that about half of them have so far never been characterized. The *kcs1* mutant is affected in wax biosynthesis and KCS1 expression in yeast leads to the production of C20 and C22 VLCFA (Todd and Post-Beittenmiller, 1999; Trenkamp et al., 2004; Tresch et al., 2012). *KCS2* and *KCS20* are highly expressed in root endodermis and the corresponding *kcs2* and *kcs20* single mutants showed a reduction of the amount of C22 monomers in suberin in accordance with yeast heterologous expression results (Trenkamp et al., 2004; Paul et al., 2006; Franke et al., 2009; Lee et al., 2009; Tresch et al., 2012). KCS4 was recently shown to be involved in the elongation of VLCFA with more than 24 carbons during root and pollen tube growth (Kim and Lee, 2021). The *kcs6/cer6/cut1* mutant displays one of the most severe waxless phenotypes, and KCS6 (like its paralog KCS5) was shown to lead to the production of C26 and C28 VLCFA in yeast (Millar et al., 1999; Hooker et al., 2002; Trenkamp et al., 2004; Tresch et al., 2012; Haslam et al., 2015; Huang et al, 2022). Acyl-CoA profiling of *kcs9* roots showed a slight accumulation of 22:0-CoA species (Kim et al., 2013), in accordance with the KCS9 specific activity deduced from *in vitro* yeast assays (Paul et al., 2006). KCS16 was shown to be involved in the biosynthesis of C36 and C38 VLCFA for wax production in leaf trichomes and pavement cells (Hegebarth et al., 2017), as well as to produce C22 and C24 VLCFA important for lateral root development (Lv et al., 2021). In agreement with the seed specific expression of *KCS18/FAE1*, heterologous expression of KCS18 in yeast leads to the accumulation of saturated and monounsaturated C20 and C22 VLCFA (James et al., 1995; Trenkamp et al., 2004; Tresch et al., 2012). The absence of a detectable KCS activity in yeast for the remaining enzymes could result from the absence or unavailability of their physiological substrates, an inadequate cell environment, competition with the endogenous yeast condensing enzymes or a poor interaction of plant KCS with the other components of the yeast FAE complex. In this study, to address these challenges and explore the functional redundancy of Arabidopsis KCS, we have engineered a novel yeast platform dedicated to the characterization of Arabidopsis VLCFA elongation. Each of the 21 KCS was expressed in this novel platform as well as in tobacco epidermal cells thus providing the first exhaustive and comparative analysis of the whole Arabidopsis KCS multigenic family.

## Materials and methods

### Plant material and growth conditions

*Arabidopsis thaliana* (L.) Heynh (ecotype Columbia–0) was used in all experiments. Arabidopsis plants were grown under controlled conditions as previously described (Joubès et al., 2008). For subcellular localization studies, *Nicotiana benthamiana* plants cultivated in controlled conditions (16 h light photoperiod, 25°C) were analyzed 2 and 5 days after agroinfiltration for subcellular localization experiments and for overexpression, respectively as previously described (Perraki et al., 2012).

### DNA, RNA and cDNA preparation, and RT-PCR analysis

Genomic DNA was extracted from Arabidopsis leaves with the DNeasy Plant kit (Qiagen, Courtaboeuf, France) and RNA from Arabidopsis tissues or yeast cells with the RNeasy Plant mini kit (Qiagen). Purified RNA was treated with DNase I using a DNA-free kit (Ambion, Austin, TX). First-strand cDNA was prepared from 1 µg of total RNA with the Superscript RT II kit (Invitrogen, Carlsbad, California) and oligo(dT)_18_ according to the manufacturer’s instructions. Semi-quantitative RT-PCR analysis and PCR on genomic DNA were performed using Q5^®^ High-Fidelity DNA Polymerase (New England Biolabs, Evry, France). PCR amplifications were performed with gene-specific primers listed in **Supplemental Table S1**.

### DNA constructs

Open reading frames (ORFs) were amplified from Arabidopsis genomic DNA and from Arabidopsis tissues cDNA using primers listed in **Supplemental Table S1**. The corresponding PCR fragments were inserted into the pDONR™;221 ENTRY vector using the GATEWAY^®^ recombinational cloning technology (Invitrogen) and subsequently transferred into pK7WG2D, pK7YWG2 and pvtLEU DESTINATION vectors (Karimi et al, 2002; Dittrich-Domergue et al., 2014) by using LR clonase II. DNA constructs transferred into the *Agrobacterium tumefaciens* GV3101 strain were used for transient expression in *N. benthamiana* leaves (Perraki et al., 2012). *Saccharomyces cerevisiae* cells were transformed with different constructs the standard lithium acetate transformation protocol (Gietz et al., 1995) and grown on minimal medium lacking appropriate amino acids.

### Yeast genetics

The coding sequences of the Arabidopsis genes *KCR*, *PAS2/HCD* and *ECR* were introduced in the yeast genome to replace the coding sequences of the following genes: *IFA38* (*YBR159W*), *PHS1*(*YJL097W*) and *Tsc13*(*YDL015C*), respectively, using the CRISPR-Cas9 method. Primers KCR1/KCR2, PAS1/PAS2 and ECR1/ECR2 (**Supplemental Table S1**) containing the gRNA sequences were cloned in the pgRNA.AarI (modified by Tsarmpopoulos et al. (2016) from the p426-SNR52p-gRNA plasmid) to obtain pgRNA.IFA38, pgRNA.PHS1 and pgRNA.TSC13. The recombination templates were obtained by PCR using primers KCR5/KCR6, PAS5/PAS6 or ECR5/ECR6 (**Supplemental Table S1**) and Arabidopsis cDNA as DNA template. Yeast cells were transformed using the standard lithium acetate transformation protocol (Gietz et al., 1995). Approximately 200 ng of plasmid p414-TEF 1p-Cas9-CYC1t (DiCarlo et al., 2013) was used to transform yeast strain InvSc1 [*MATa his3Δ1 leu2 trp1-289 ura3-52 MATα his3Δ1 leu2 trp1-289 ura3-52*] to obtain yeast strain InvSc1-Cas9. Allelic exchanges were realized by iterative approach, the yeast strain InvSc1-Cas9 was transformed with 200 ng of plasmid pgRNA.IFA38, pgRNA.PHS1 or pgRNA.TSC13 and 1 nmol of corresponding recombination templates for homologous recombination. After transformation, cells were plated on selective medium. Loss of pgRNA plasmid was realized between two transformations. The TRIPLE strain resulting of these allelic exchanges was verified by PCR and sequenced using KCR7/KCR8, PAS7/PAS8 and ECR7/ECR8 primers (**Supplemental Table S1**). For deletion of the *ELO3* gene, primers ELO1/ELO2 (**Supplemental Table S1**) containing the gRNA sequences were cloned into pgRNA.AarI vector to obtain pgRNA.ELO3 vector, the yeast strain TRIPLE was transformed with 200 ng of plasmid pgRNA.ELO3 and 1 nmol of ELO5 and ELO6 annealed primers (**Supplemental Table S1**) as template for homologous recombination. Inactivation was verified by PCR and sequencing using ELO7/ELO8 primers (**Supplemental Table S1**). DNA target of Cas9 were determined on http://crispr.dfci.harvard.edu/SSC/ . For strain growth analysis, strains were grown using a FLUOstar Omega microplate reader (BMG LABTECH, Champigy sur Marne, France), maintained at 30˚C and growth was monitored by measuring OD600 every 20 minutes.

### Lipid analyses

For fatty acyl chain analyses in yeast, yeasts were grown in 4 mL of appropriate liquid minimal medium. Yeast cells grown for 72 hours at 30°C were pelleted and washed in 2 mL of water. Fatty acid methyl esters were obtained by transmethylation at 85°C for 2 hours. For this, yeast cell pellets were resuspended in 1 mL 0.5 M sulphuric acid in methanol containing 2% (v/v) dimethoxypropane and 20 μg of heptadecanoic acid as internal standard. After cooling, 1 mL of 2.5% (w/v) NaCl was added, and fatty acyl chains were extracted in 300 µL of hexane. For fatty acyl chain analyses in N*. benthamiana*, three agroinfiltrated leaf discs, 5 days post agroinfiltration, were transferred into tubes containing 1 mL 0.5 M sulphuric acid in methanol containing 2% (v/v) dimethoxypropane and 20 μg of heptadecanoic acid as internal standard. Transmethylation was performed at 85°C for 2 hours. After cooling, 1 mL of 2.5% (w/v) NaCl was added, and fatty acyl chains were extracted in 300 µL of hexane for analysis using an Agilent 7890A gas chromatograph equipped with an HP-5MS column (30 m x 0.25 mm x 0.25 mm) and a flame ionization detector. The initial temperature of 50°C was held for 1 min, increased at 25°C.min^-1^ to 150°C, held for 2 min at 150°C, increased again at 10°C.min^-1^ to 320°C, and held for 6 min at 320°C. Qualitative analyses were performed using an Agilent 7890A gas chromatograph equipped with an HP-5MS column and an Agilent 5975 mass spectrometric detector (70 eV, mass-to-charge ratio 50–750). The same GC program was used, with helium (1.5 mL.min^-1^) as carrier gas. Acyl chains quantities were determined relatively to the intensity of the pic associated to the quantity of heptadecanoic acid.

### Acyl-CoA analysis

Replicated yeast samples (2 OD) were frozen in liquid nitrogen and stored at -80°C prior to extraction. Samples were extracted after Haslam and Larson (2021) for reverse-phase liquid chromatography (LC) with electrospray ionization tandem mass spectrometry (multiple reaction monitoring: using a SCIEX 4000QTRAP instrument) in positive ion mode. LC-Mass Spectrometry/Mass Spectrometry (Multiple Reaction Monitoring) analysis followed the methods described in Haslam and Larson (2021). Acyl-CoAs were separated using an Agilent 1200 LC system; Gemini C18 column, 2 mm inner diameter, 150 mm with 5 µm particles. For the purpose of identification and quantification, standard acyl-CoA esters with chain lengths from C14 to C20 were synthesized from free acids or lithium salts (Sigma-Aldrich, St. Louis, MO, USA). Heptadecanoyl-CoA (ammonium salt) was used as an internal standard in each analytical run. Retention times were also confirmed using acyl-CoA standards from Avanti.

### Confocal microscopy

Live imaging was performed using a Leica SP5 confocal laser scanning microscopy system (Leica, Wetzlar, Germany) equipped with Argon, DPSS, He-Ne lasers, hybrid detectors and 63x oil-immersion objective. Two days post agroinfiltration, *N. benthamiana* leaf samples were gently transferred between a glass slide and a cover slip in a drop of water. The plasmid ER-gk CD3-955 was used as a fluorescent ER marker (Nelson et al., 2007). YFP and GFP fluorescence were observed using excitation wavelength of 488 nm and their fluorescence emission was collected at 490–540 nm.

### Statistical analyses

PCA analyses were performed using the platform MetaboAnalyst 5.0 (https://www.metaboanalyst.ca/ ). The different dataset obtained in TRIPLE and TRIPLE *Δelo3* were processed using Log transformation (base 10) and Auto scaling. Further pairwise comparisons reported in yeast assays were performed using a Tukey *post hoc* test. FAMEs profiles obtained after transient expression of KCS enzymes in *Nicotiana benthamiana* were compared using a Wilcoxon test, the P value was adjusted using the Bonferroni method. Apart from the PCA analysis, all the statistics were performed with RStudio (2021.09.0 + 351).

### Accession numbers

Sequence data from this article can be found in the Arabidopsis Genome Initiative and SGD databases under the following accession numbers: *Arabidopsis thaliana*: *KCS1*, At1g01120; *KCS2*, At1g04220; *KCS3*, At1g07720; *KCS4*, At1g19440; *KCS5*, At1g25450; *KCS6*, At1g68530; *KCS7*, At1g71160; *KCS8*, At2g15090; *KCS9*, At2g16280; *KCS10*, At2g26250; *KCS11*, At2g26640; *KCS12*, At2g28630; *KCS13*, At2g46720; *KCS14*, At3g10280; *KCS15*, At3g52160; *KCS16*, At4g34250; *KCS17*, At4g34510; *KCS18*, At4g34520; *KCS19*, At5g04530; *KCS20*, At5g43760; *KCS21*, At5g49070; *KCR*, At1g67730; *PAS2/HCD*, At5g10480; *ECR*, At3g55360; *ACT2*, At1g49240; *Saccharomyces cerevisiae: ACT1*, YFL039C; *ELO3*, YLR372W; *IFA38*, YBR159W; *PHS1*: YJL097W; *TSC13*: YDL015C.

## Results

### Engineering a novel yeast system to explore the catalytic activities of Arabidopsis KCS

**Figure 1:**
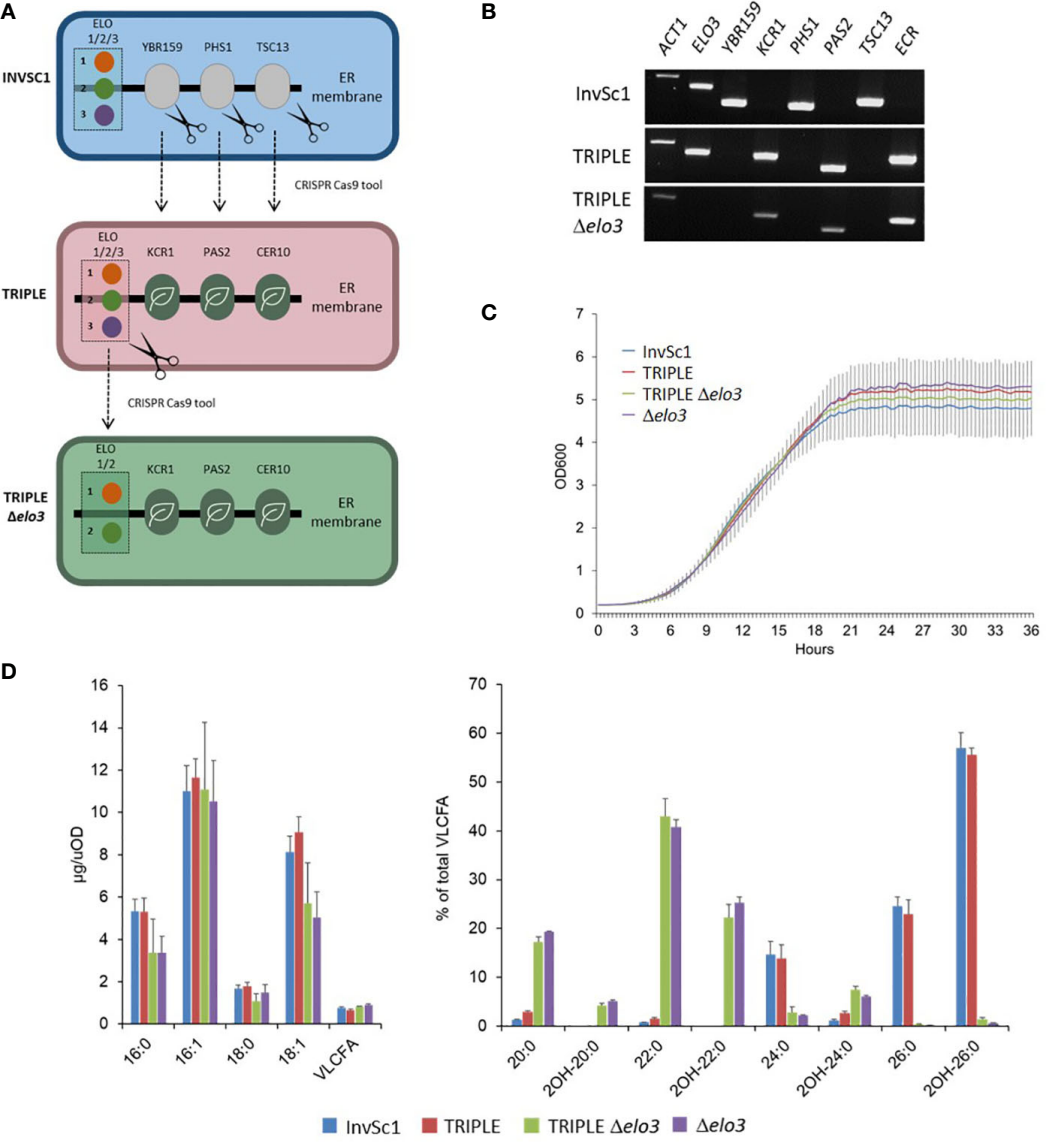
Genetic complementation of yeast elongase by Arabidopsis components. CRISPR Cas9 technology was used to replace the coding sequences of the yeast last three enzymes involved in fatty acid elongation (YBR159, PHS1 and TCS13) by their plant orthologues (KCR, HCD/PAS2 and ECR/CER10) and to delete the *ELO3* gene encoding the enzyme which elongates C22:0 to C24:0 and C26:0. **(A)**. Schematic of the strategy. **(B)**. Analysis of gene expression by RT-PCR in the modified TRIPLE and TRIPLE Δ*elo3* strains compared to non-modified InvSc1 strain. *ACT1* transcripts are used as a loading control. **(C)**. Growth analysis of InvSc1, TRIPLE, TRIPLE Δ*elo3* and Δ*elo3* strains. Values are the average of ten samples. **(D)**. Comparison of the FAMEs profile of InvSc1 control strain with the FAMEs profiles of the TRIPLE, TRIPLE Δ*elo3* and Δ*elo3* strains grown for 72 hours. Mean values (µg/uOD or percentage of total VLCFA) are given with SD (n = 5). Each FAME species is designed by carbon chain length and degree of unsaturation.

Yeast and plant microsomal FAE complexes are very similar and composed of four core subunits catalyzing identical reactions. Genetic analyses and complementation assays emphasized the strong homology of yeast HCR, HCD and ECR subunits (YBR159, PHS1 and TSC13) with the corresponding plant enzymes. In contrast, the condensing enzymes of the yeast complex, i.e. the ELO proteins ELO1/2/3, share no sequence homology with plant KCS enzymes. Heterologous expression of some Arabidopsis KCS could rescue the lethality of *elop* family lethal multiple mutants, showing that at least some plant condensing enzymes can form functional elongase complexes with the yeast core subunits to restore elongase activity. Yet, as mentioned before, only nine Arabidopsis KCS showed an activity when expressed in yeast, suggesting that WT yeast cells do not provide a suitable environment for the activity of the twelve remaining enzymes. Based on these previous results we hypothesized that these twelve KCS could not, or not efficiently, form a complex with the yeast HCR, HCD and ECR subunits. To better study the catalytic activity of plant FAE complexes, we engineered a yeast platform to reconstitute whole Arabidopsis complexes and study the chain length specificity of each Arabidopsis KCS enzyme. First, using the CRISPR-Cas9 genome editing tool, we designed a yeast strain named TRIPLE, in which the coding sequence for yeast endogenous FAE core subunits YBR159, PHS1 and TSC13 were replaced by the corresponding Arabidopsis genes *KCR1*, *PAS2* and *CER10* (**Figures 1A, B**). Both strains display similar growth curves (**Figure 1C**) and the TRIPLE strain shows a fatty acid profile similar to the wild-type InvSc1 strain with a strong accumulation of C16 and C18 long chain fatty acids followed by the elongation of VLCFA up to C26 (**Figure 1D**). This result confirmed that the last three plant core subunits complement the yeast endogenous enzymes and formed functional FAE complexes with the yeast ELO proteins. An additional and/or alternative possibility accounting for the absence of activity for twelve out of 21 Arabidopsis KCS in yeast is that they could be outcompeted by that of the yeast endogenous ELO proteins responsible for the elongation of fatty acids from C14 to C26. We thus designed a second yeast strain named TRIPLE Δ*elo3* in which ELO3, which is involved in the endogenous elongation of C22 to C24 and C26 VLCFA, was deleted (**Figures 1A, B**). In this strain, the microsomal FAE complex is unable to elongate VLCFA beyond C22 which accumulated together with C20. The fatty acid profile of the TRIPLE Δ*elo3* strain is similar to the Δ*elo3* strain profile and both strains show similar growth curves (**Figures 1C, D**).

### Heterologous expression of Arabidopsis KCS in yeast

**Figure 2:**
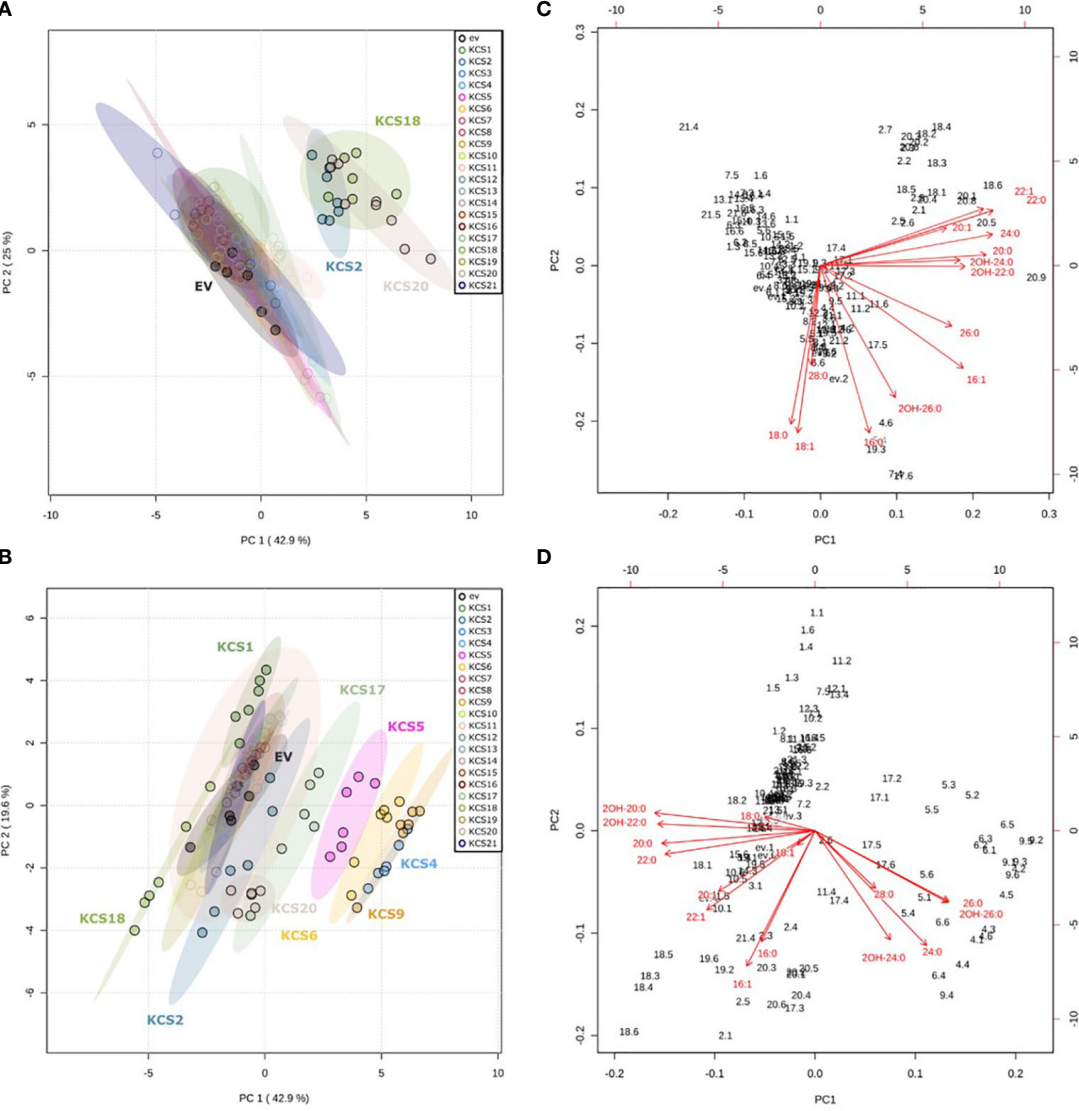
Principal component analysis (PCA) of the effect of KCS enzymes on FAMEs profiles after expression in InvSc1 TRIPLE and TRIPLE *Δelo3* strains. Scores obtained for the two first principal components PC1 and PC2 in **(A)** InvSc1 TRIPLE and **(B)** InvSc1 TRIPLE *Δelo3*. Corresponding biplots in **(C)** InvSc1 TRIPLE and **(D)** InvSc1 TRIPLE *Δelo3*. The PCA analyses are of data presented in **Figure 3**.

The 21 Arabidopsis KCS genes were cloned and expressed under the transcriptional control of the constitutive ADH1 promoter in the TRIPLE and TRIPLE Δelo3 *strains (* **Supplemental Figure S1**) and fatty acids were extracted, methylated and analyzed by GC-MS and GC-FID for identification and quantification, respectively. To visualize the changes in VLCFA contents in the 42 strains expressing Arabidopsis KCS genes, PCAs were performed based on the relative quantification of FAMEs from C16 to C28. In the 21 AtKCS-expressing TRIPLE strains, three AtKCS (KCS2, KCS18 and KCS20) separated from the control strain (empty vector, ev), the first two principal components representing 68% of the total variance (**Figure 2A**). In the AtKCS-expressing TRIPLE Δ*elo3* strains, six other AtKCS (KCS1, KCS4, KCS5, KCS6, KCS9 and KCS17) differentiated from the control strain (empty vector, ev), PC1 and PC2 representing 62.5% of the total variance (**Figure 2B**). The corresponding biplots indicated that C20, C22 and C24 products were upregulated in the AtKCS-expressing TRIPLE strains along the first principal component (**Figure 2C**). Conversely, C20 and C22 products were down regulated and C24 and C26 compounds upregulated in the AtKCS-expressing TRIPLE Δ*elo3* strains (**Figure 2D**). Therefore, the use of the TRIPLE strain highlighted the biochemical function of AtKCS specifically elongating C18 into C20 up to C24 acyl chains whereas the TRIPLE Δelo3 strain appeared more suitable for the biochemical characterization of AtKCS accumulating C24 to C26 compounds.

**Figure 3:**
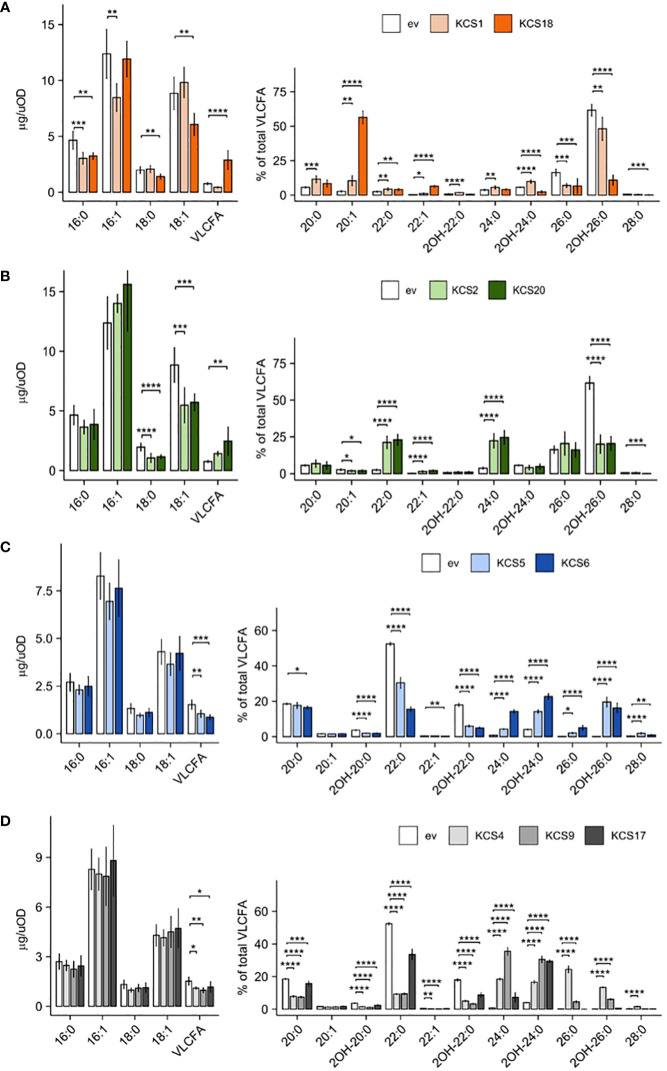
Expression of AtKCS in yeast triggers an over-production of VLCFA. Comparison of FAMEs profiles of TRIPLE strain (transformed with empty vector, ev) and TRIPLE transformed with KCS1 and 18 **(A)**, TRIPLE transformed with KCS2 and 20 **(B)**. Comparison of FAMEs profiles of TRIPLE *Δelo3* strain (transformed with empty vector, ev) and TRIPLE *Δelo3* transformed with KCS5 and 6 **(C)**, TRIPLE *Δelo3* transformed with KCS4, 9 and 17 **(D)**. Mean values (µg/uOD or percentage of total VLCFA) are given with SD (n = 6). Each FAME species is designated by carbon chain length and degree of unsaturation. Significance was determined with a Tukey *post hoc* test (*P < 0.05, **P < 0.01, ***P < 0.001, ****P < 1e^-4^).

Overall, we found nine functional AtKCS in our novel yeast heterologous platforms (KCS1, 2, 4, 5, 6, 9, 17, 18, 20; **Figure 3**) while the expression of the twelve other AtKCS (KCS3, 7, 8, 10, 11, 12, 13, 14, 15, 16, 19 and 21) showed no significant effects on VLCFA accumulation in both TRIPLE and TRIPLE Δelo3 strains (**Supplemental Table S2**). Previous phylogenetic analysis showed that Arabidopsis KCS proteins form eight subclasses and identified eight pairs of paralogous proteins (KCS2-KCS20, KCS5-KCS6, KCS9-KCS17, KCS3-KCS12, KCS8-KCS16, KCS13-KCS14, KCS10-KCS15 and KCS7-KCS21; Joubès et al., 2008). In the present comparative analysis, we observed that functional AtKCS showing a close sequence identity display similar catalytic activity. Three pairs of paralogs over the eight (KCS2-KCS20, KCS5-KCS6 and KCS9-KCS17) were functional in the yeast systems. In the TRIPLE strain, the expression of KCS2 or KCS20, which share 85% identity, led to a strong accumulation of C22 and C24 saturated acyl chains (**Figure 3B**), demonstrating that those enzymes are responsible for the elongation of C20 into C22 and C22 to C24 compounds. KCS2 and KCS20 form the subclass ζ with KCS11 which shares 71% and 70% identity with KCS2 and KCS20, respectively. Thus, the paralogs KCS2 and KCS20 share overall a common substrate selectivity for C20 acyl chains. In the TRIPLE Δelo3 strain, KCS5 and KCS6, which share 88% identity, led to the elongation of C22 into C24 up to C28 compounds (**Figure 3C**). Unlike the two other pairs of paralogs, KCS9 and KCS17, which share 81% identity, displayed different elongation specificities in the TRIPLE Δelo3 strain. Besides both KCS9 and KCS17 being able to perform the elongation of C22 saturated acyl chains into C24 products, only KCS9 can produce C26 VLCFA (**Figure 3D**). Together with those two KCS, KCS4 shares a relatively high identity, 75% with KCS9 and 70% with KCS17, and form the subclass α. The expression of KCS4 in the TRIPLE Δelo3 strain led to even higher amounts of C26 compounds compared to KCS9 (**Figure 3D**). Therefore, the three enzymes of the subgroup α appear to share the ability to use C22 saturated acyl chains as initial substrate to elongate them into C24 and C26 VLCFA. However, each enzyme performed those elongation steps in different ratios. Among the other phylogenic subclasses, only two single enzymes were found functional in our system; in each case, the enzyme showed an outlying substrate specificity compared to that of the other subgroups described above. KCS1 from the subclass β was found to elongate C16 acyl chains into C18 up to C22 products in the TRIPLE strain, while KCS18 from the subclass δ displayed a unique substrate specificity with a very strong accumulation of monounsaturated C20 acyl chains (**Figure 3A** ***).***

**Figure 4:**
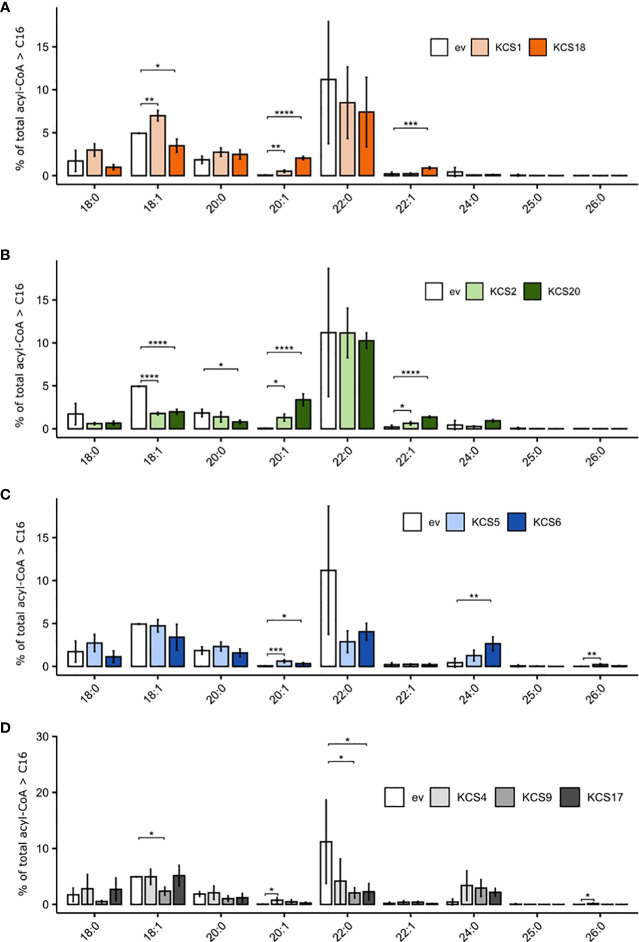
Comparison of acyl-CoA profiles of TRIPLE Δ*elo3* strain (transformed with empty vector, ev) and TRIPLE Δ*elo3* strain transformed with different AtKCS. **(A)** KCS1 and 18 **(B)** KCS2 and 20 **(C)**. KCS5 and 6 **(D)** KCS4, 9 and 17. Mean values (percentage of total acyl-CoA > or = C16) are given with SD (n = 3 to 5). Each acyl-CoA species is designated by carbon chain length and degree of unsaturation. Significance was determined with a Tukey *post hoc* test (*P < 0.05, **P < 0.01, ***P < 0.001, ****P < 1e^-4^).

VLCFA metabolism was further investigated by profiling the acyl-CoA pools of the TRIPLE Δelo3 yeast strain expressing functional AtKCS for 16 hours using liquid chromatography (electrospray ionization-tandem) mass spectrometry and multiple reaction monitoring (MRM). The acyl-CoA pool was usually dominated by C16 species, which represented 80.4 ± 3.6% of the total, while <C16-CoAs were minor (4.3 ± 0.9% of the total; **Supplemental Table S3**). A closer look at ≥ C18 molecular species (**Figure 4**) showed that, in comparison to the control strain expressing an empty vector, the expression of KCS1 increased the levels of 18:1-and 20:1-CoAs, while that of KCS18 led to increases in the levels 18:1-, 20:1- and 22:1-CoAs (**Figure 4A**). Expression of the subclass ζ AtKCS (KCS2 and 20) had a low impact on C22:0-CoA, but slightly increased the levels of 20:1- and 22:1-CoAs whereas the levels of 18:1-CoA decreased (**Figure 4B**). In contrast, AtKCS from clade α (KCS4, 9 and 17) and γ (KCS5 and 6) resulted in high decreases in C22:0-CoA and strong increases in C24:0-CoA levels (**Figures 4C, D**). In addition, expressing KCS4, 5 and 6 significantly increased the levels of 26:0-CoA. Globally, these analyses showed modifications of the acyl-CoA profiles consistent with the changes observed in the FAMEs profiles *(* **Figure 3** *).*

### Expression of Arabidopsis KCS in *Nicotiana benthamiana*

**Figure 5:**
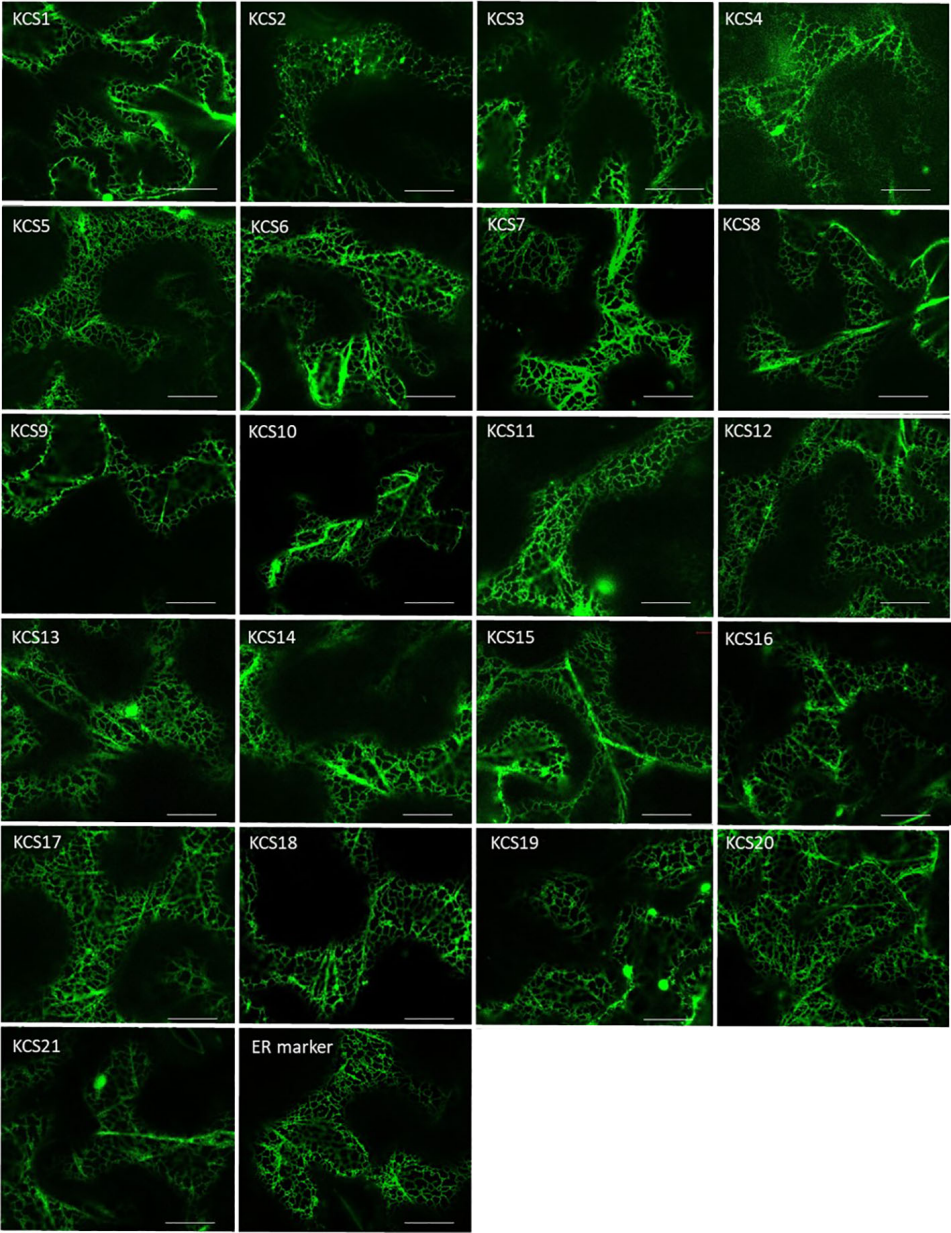
The 21 Arabidopsis KCSs localize in the endoplasmic reticulum of tobacco cells. Confocal images of *Nicotiana benthamiana* leaves transiently transformed with KCS-YFP constructs. The 21 Arabidopsis KCS ORFs have been cloned into the pK7YWG2 destination vector, transferred into the Agrobacterium tumefaciens strain GV3101 and used for transient expression in leaves. The plasmid ER-gk CD3-955 was used as an ER marker. Bars = 20µm.

The use of our engineered yeast platform enabled the characterization of 9 out of 21 AtKCS. To further analyze their *substrate specificities* and to explore that of the remaining KCS in a homologous system, we transiently expressed the 21 KCS in the epidermal cells of *N. benthamiana*. First, we checked their subcellular distribution by generating fluorescent fusion proteins of AtKCS fused to the YFP at their C-terminus. For each protein, similar subcellular locations were obtained. As shown in **Figure 5**, we found the AtKCS-YFP fusion proteins to label a polygonal and tubular network characteristic of the endoplasmic reticulum (ER). Any significant labelling in any other membranes for all the tested constructs was found, demonstrating that the different AtKCS-YFP fusion proteins were correctly targeted to the ER.

**Figure 6:**
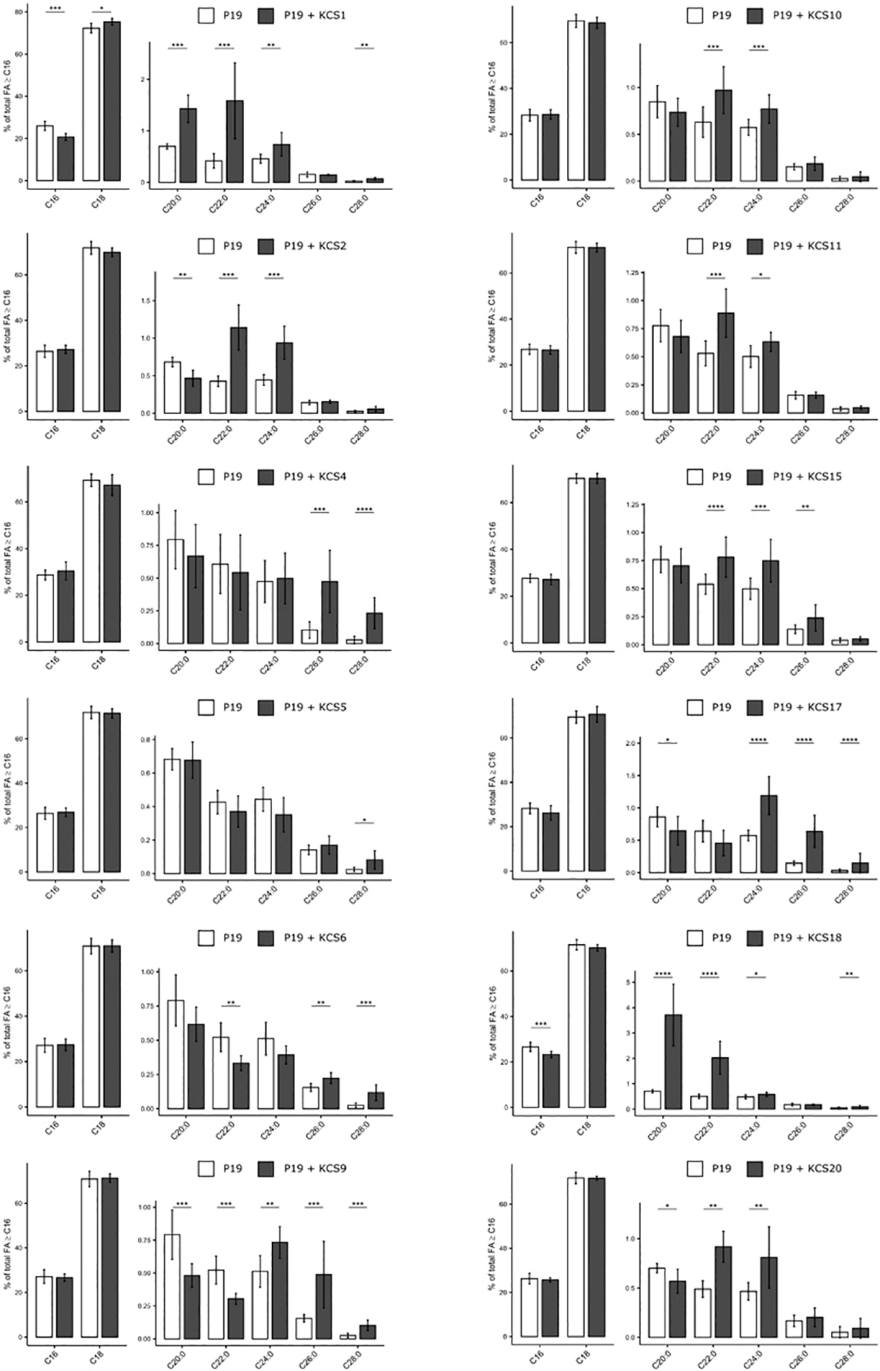
Heterologous expression of 12 AtKCS impacts FAMEs composition of *Nicotiana benthamiana* leaves. Comparison of FAMEs profiles obtained in leaves co-expressing KCS and the gene silencing suppressor P19 with the control condition (leaves transformed with P19 only). Mean values (percentage of total FA > or = C16) are given with SD (n>6). Significant changes were determined through a Wilcoxon test (*P < 0.05, **P < 0.01, ***P < 0.001, ****P < 1e^-4^).

Second, we performed fatty acid transmethylations on the transformed leaves and compared the long chain and very-long chain fatty acid profiles from C16 to C28 acyl chains. Overall, twelve AtKCS displayed a functional activity in N. benthamiana (**Figure 6**). The nine AtKCS previously characterized in TRIPLE and TRIPLE Δelo3 strains were functional in N. benthamiana and showed FAMEs profiles consistent with the FAMEs profiles described in yeast. Altogether, their expression affected the production of VLCFA from C20 to C28. The heterologous expression of KCS1 led to the increase of C18 up to C24 acyl chains at the expense of C16. A higher accumulation of C20:1 and C22:1 was observed with the ectopic expression of KCS18, which displays a strong affinity for monounsaturated substrates. KCS2 and KCS20 led to an increase of C22 and C24 acyl chains. The ectopic expression of KCS17, KCS9 and KCS4 led to a higher accumulation of C24 to C28 compounds whereas C26 and C28 acyl-chains were increased upon KCS6 expression. Its paralog KCS5 also led to the greater accumulation of C28 compounds, however its activity in planta was not as significant as in the yeast system. KCS1, KCS9 and KCS17 led to an increase of longer compounds compared to FAMEs profiles described in the yeast system. Those results imply that N. benthamiana endogenous FAE complexes use the products generated by AtKCS-containing FAE complexes to perform further elongation steps. In addition to the AtKCS already described in the yeast, the ectopic expression of KCS10, KCS11 and KCS15 induced significant changes in the FAMEs profiles of N. benthamiana leaves. This suggests that these enzymes require co-factors not found in yeast cells that are essential for their activities. The expression of KCS11 led to a slighter accumulation of C22 and C24. KCS11 forms the subclass ζ with KCS2 and KCS20 which produced C22 and C24 VLCFA in yeast (**Figure 3B**). Thus, the three enzymes of the subgroup ζ may share a similar substrate selectivity for C20 acyl chains. KCS10 and KCS15 form the subclass ϵ as paralogs and share 51% identity (Joubès et al., 2008). In N. benthamiana, they similarly increased C22 and C24 acyl-chain contents, but only KCS15 led to an increase in C26. The expression of the other AtKCS (KCS3, 7, 8, 12, 13, 14, 16, 19 and 21) had no significant effects on VLCFA accumulation in N. benthamiana leaves *(* **Supplemental Figure S2** *).*

## Discussion

The current view of fatty acid elongation in plants is that multiple elongase complexes perform sequential and parallel reactions to produce the broad chain-length-range of VLCFAs found in plants, and that the condensing enzymes dictate spatial and substrate specificity of each elongation complex (Batsale et al., 2021). If we consider that each KCS-containing FAE complex is able on average to lengthen the carbon chain by four carbon atoms (two cycles of elongation), the number of copies of KCS genes present in plant genomes, 21 in Arabidopis (Joubès et al., 2008), 26 in maize (Campbell et al., 2019), 28 in Medicago (Chai et al., 2021), 30 in peanut (Huai et al., 2020), and 58 in cotton and rapeseed (Xiao et al., 2016; Xue et al., 2020), largely exceeds the minimum number of KCS needed to produce all of the chain lengths found in plant VLCFA (*i.e.* from 20 up to 38 carbon atoms). Furthermore, studies conducted in Arabidopsis have clearly shown functional redundancy as well as broad spatiotemporal expression for many KCS. Indeed, more than half of Arabidopsis KCS genes are ubiquitously or near-ubiquitously expressed throughout plant tissues, while the others show expression patterns mainly restricted to reproductive organs (Joubès et al., 2008). This implies a complex regulation of KCS gene expression in the different tissues and throughout plant development, and may explain why most Arabidopsis *kcs* single mutants show no obvious phenotype. In addition, as VLCFA are incorporated into several lipid pools (phospholipids, sphingolipids or surface lipids), alterations in VLCFA levels have pleiotropic consequences, which greatly complicates unravelling the exact role of each KCS in VLCFA biosynthesis. In this study, the full set of Arabidopsis KCS genes was expressed in novel expression platforms in order to provide an exhaustive characterization of their biochemical activities and provide knowledge towards the characterization of their function in Arabidopsis.

### Overcoming challenges to characterize novel Arabidopsis KCS

Because the yeast and plant FAE complexes are very similar, several AtKCS had previously been shown to be active in yeast (Trenkamp et al, 2004; Paul et al., 2006; Blacklock, 2006; Tresch et al., 2012). Tresch et al, 2012 expressed 17 AtKCS genes in wild-type yeast and *elo2* or *elo3* single mutants and confirmed the activity of seven of them: KCS1 and KCS18, which produced saturated and monounsaturated C20 and C22 VLCFA; KCS2 and KCS20, producing C20, C22 and C24 VLCFA; KCS5 and KCS6, mainly producing C24 to C28 VLCFA; and KCS17, producing C24 VLCFA. A similar strategy was recently used to study maize (*Zea mays*) FAE complexes unravelling the activity of 5 out of the 26 ZmKCS tested (Campbell et al., 2019; Stenback et al., 2022). Expression of none of these *ZmKCS* genes affected the VLCFA profiles of wild-type, *elo2* or *elo3* single mutants, but five of them were capable of complementing the *elo2 elo3* double mutant, and showed novel VLCFA profiles reflecting their different catalytic specificities. ZmKCS2 produced C20 and C22 VLCFA, ZmKCS4, ZmKCS11 and ZmKCS20 produced VLCFA up to C24, while ZmKCS15 could elongated VLCFA up to C26. Interestingly, ZmKCS2, 4, 11 and 20 are homologous to Arabidopsis KCS 2, 4, 11 and 20, while ZmKCS15 is rather similar to AtKCS4. In addition to *in vivo* studies, the yeast system was also used for *in vitro* assays of elongation/condensation activity using microsomal membranes. This approach confirmed the chain length specificity of KCS1, 2, 18 and 20, and revealed the capacity of KCS9 to produce C22 and C24 VLCFA (Blacklock, 2006; Paul et al., 2006). Using the same strategy, Blacklock (2006) were unable to unravel the activity of several KCS tested (KCS3, 4, 7, 16 and 19) and showed only very minor differences in the yeast fatty acid profiles when expressing KCS11 and 17, whose chain length specificities remained nevertheless unclear.

In order to provide an exhaustive and complete description of Arabidopsis KCS we set up a novel analytic platform enabling the analyses of plant KCS activities by combining and comparing their heterologous expression in yeast and in planta. First, to provide an optimal environment for AtKCS activities in yeast, we reconstituted whole Arabidopsis FAE complex in place of the endogenous yeast FAE. Further, to avoid competition with the yeast endogenous FAE activity, we deleted the condensing enzyme ELO3 in our engineered strain. Using these engineered strains, we obtained results replicating that of previous studies concerning KCS1, KCS18, KCS2 and KCS20, KCS5 and KCS6 and KCS9 and KCS17. In addition, our strategy clearly described the activity of KCS11, which we found producing C20, C22, C24 and C26 VLCFA, and enabled the first heterologous characterization of KCS4, which mainly produced C24 and C26 VLCFA in the TRIPLE Δ*elo3* yeast strain. Since Tresch et al. (2012) could not observe any change in yeast VLCFA when expressing AtKCS4 with the three other core subunits from yeast FAE complexes (YBR159, PHS1 and TSC13), these results suggest that AtKCS4 could not efficiently interact with them. In contrast, a better interaction/collaboration with the plant subunits KCR1, PAS2 and CER10 likely supports strong AtKCS4 activity. Similarly, Stenback et al. (2022) hypothesized that most ZmKCS could not complement yeast mutant because of interaction issues between ZmKCS and the other core subunits of the yeast FAE system. Second, to palliate the potential lack of appropriate substrate in yeast cells and provide a close to native cell/membrane environment, we expressed AtKCS in the epidermis of *N. Benthamiana*. This strategy enabled the first characterization of KCS11, which we found producing C20, C22, C24 and C26 VLCFA, and of KCS10 and KCS15 expression of which promoted the accumulation of C22-C24 and C22-C26 levels, respectively. Given that the substrate specificity for these three enzymes (C20-C24) and their availability in yeast cells, these results suggest that KCS10, KCS11 and KCS15 may require for their activities obligatory plant-specific cofactors whose identity should be the focus of future research. The functional characterization of KCS10 and KCS15 supports previous data obtained in plants. The functional characterization of KCS10 and KCS15 support previous data obtained in plants. Indeed, the phenotype of the *fiddlehead* (*kcs10*) mutant, which is characterized by fusion events between reproductive organs and/or leaves as well as by two times fewer trichomes on leaves, suggests that KCS10 products regulate the differentiation of protoderm cells into trichomes, whose correct development is crucial for preventing organ fusions (Yephremov et al., 1999; Pruitt et al., 2000). Our data suggest that C22 and C24 VLCFA produced by KCS10, or derivatives, might be involved in this process. Similarly, Zhang et al. (2021) recently showed that three AtKCS genes expressed in tapetal cells at anther stages 8-10 are involved in pollen coat lipid accumulation. Indeed, cellular observation and lipid staining of the Arabidopsis *kcs7/15/21* triple mutant showed that a decrease of the pollen coat lipids leads to delayed pollen grain hydratation and pollen tube growth. However, the lack of pollen coat lipid analysis in this study made it unclear which VLCFAs might be affected in this triple mutant. These results nevertheless suggest that these three KCS are functional and have overlapping roles in pollen maturation, while our data indicate that C22 to C26 VLCFA, or derivatives, might be involved in pollen coat lipid synthesis. In contrast, we were not able to show any activity for KCS13, although the phenotype of the *kcs13* mutant, which exhibits a much higher stomatal density at elevated CO_2_, suggests a role of KCS13 in stomatal cell fate (Gray et al., 2000). Since the products generated by KCS13 remain unknown, the involvement of VLCFA or derivatives in this process still needs to be demonstrated. Similarly, Chai et al. (2021) recently characterized in *Medicago truncatula* a seed coat-specific KCS, KCS12, which is a close ortholog of the uncharacterized Arabidopsis KCS12. They showed that *kcs12* mutant seeds lost physical dormancy, and that the amount of C24 lipid polyester monomers is decreased in the seed coat, suggesting that KCS12 is an active enzyme in seeds. The expression of AtKCS12 in yeast and tobacco in this study does not suggest a similar role in Arabidopsis, and the AtKCS responsibles for C24 seed coat suberin components still remain to be characterized. Finally, we also could not show any activity for KCS16 although Hegebarth et al. (2017), using heterologous expression in yeast, showed that AtKCS16 produces VLCFA up to 38 carbons. Demonstrating such an activity was only possible by co-expressing KCS16 with KCS6 and CER2-LIKE1, which most probably provided the required C34 acyl-CoAs substrates. More recently, results from Lv et al. (2021) suggest that AtKCS16 could also produce C22 and C24 VLCFA to support lateral root development. Nevertheless, we could not show such an activity in any of our platforms. The fact that the same KCS could be involved in the synthesis of VLCFA with such different chain lengths is quite puzzling and not consistent with most results published so far.

### Other substrates, partners, or functions: what is missing?

If our results show that the heterologous expression of twelve Arabidopsis KCS affected the VLCFA profiles of yeast and/or tobacco leaves, our strategy did not allow for describing a potential activity for nine AtKCS. In our yeast platforms, KCS formed FAE complexes together with the Arabidopsis KCR1, PAS2 and CER10 enzymes, so that we can refute that weak interactions between plant KCS and the other FAE core subunits would impeach their activity. In agreement with *in silico* models suggesting that KCS can form dimers (Gonzalez-Mellado et al., 2019), Kim et al. (2022) recently showed using yeast two-hybrid and bimolecular fluorescence complementation assays that homo- and hetero-dimerization occur between KCS2, KCS6 and KCS9. These results suggest that FAE complexes might contain several KCS and that KCS can form heterodimers. It is therefore possible that certain KCS only function as heterodimers, a hypothesis which should be tested in future studies.

Although it has long been suggested that the elongase is an ER-associated multiprotein complex (Cassagne et al., 1994; Lassner and Lardizabal, 1996), still very little is known about its quaternary structure. In addition, several proteins associate with FAE complexes and are required for the completion of the elongation process. These include the CER2-LIKE proteins which are auxiliary enzymatic components of the elongase complexes and obligatory co-factors for specific condensing enzymes, such as KCS6 and KCS5, to enable the production of VLCFA with more than 30 carbons (Haslam et al., 2015; Hegebarth et al., 2017; Wang et al., 2017; Haslam and Kunst, 2021; Gonzales-Vigil et al, 2021). Although the mode of action of these proteins remains to be elucidated, it was suggested that they stabilize FAE complexes, enhance their activity or allow the newly elongated acyl-CoA to be presented back to the KCS enzyme for additional elongation cycles. The Arabidopsis molecular chaperones PAS1 and AKR2A proteins, which are known to target protein complexes and regulate their assembly or activity, are other examples of FAE complex regulators (Roudier et al., 2010; Chen et al., 2020). The absence of such proteins, or that of other unidentified FAE regulators may be the reason why several KCS are found inactive in yeast cells.

Other possibilities for the uncharacterized AtKCS are that their preferred substrate was not present in our heterologous platforms, or that their products were not considered in our analytical pipeline. Uncharacterized KCS enzymes may act on substrates other than long chain fatty acids, use short or medium chain-length fatty acids as substrates or have their products further elongated by other KCS or modified so that global changes in fatty acid profiles are not detected. For example, methyl-branched wax compounds are important components of cuticular waxes in Arabidopsis and other species (Busta et al., 2017; Li et al., 2021). Their biosynthesis is still mostly uncharacterized, however it has been hypothesized that branched LCFAs may be metabolized by pathways analogous to those generating unbranched wax compounds (Busta and Jetter, 2017). Analyses of *kcs5* or *kcs6* waxes showed a reduced amount of both unbranched and iso-branched compounds up to C28 suggesting that both KCS accept unbranched and branched substrates (Busta and Jetter, 2017; Huang et al., 2022). Nevertheless, we cannot exclude that some yet to be characterized AtKCS enzymes specifically accept branched compounds with shorter chain lengths.

## Conclusions

Together with previous studies conducted on KCS, this work emphasized several clues based on the catalytic activity of twelve AtKCS, the biochemical and physiological roles of the nine others remain to be elucidated. First, the overall characterization performed here on the whole AtKCS family highlights the ability of the different AtKCS to cover a limited number of elongation cycles (two-three). Thanks to overlapping substrate specificities, they allow the elongation of VLCFAs from C20 to C28. Second, functional redundancies were also observed between different AtKCS generally composing a same phylogenic subclass according to the phylogeny of AtKCS proteins which are distributed into eight clades (Joubès et al., 2008). For instance, KCS2, KCS11 and KCS20 included in clade ζ, all produce C20 to C24 VLCFA, KCS9, KCS4 and KCS17, composing the clade α, produce VLCFA between C24 and C28, while KCS5 and KCS6 both produce C24 to C28 in the clade γ. Third, among AtKCS proteins characterized to date, only few isoenzymes seem to display both restricted expression patterns and functions such as KCS6 which is highly expressed in the epidermis of aerial organs or KCS18 which is specifically expressed in seeds, the condensation products of which seem to be preferentially used in the biosynthesis of cuticular waxes and TAGs, respectively. Other AtKCS, are rather broadly expressed and might be involved in various lipid biosynthetic pathways. Fourth, our data suggest that certain AtKCS may require essential co-factors (including potential homo or hetero-dimerization) or use unconventional substrates. We believe that the analytical platform developed in this study, especially the engineered yeast reconstituting the complete Arabidopsis FAE complexes, provides an efficient tool to address these questions in future research seeking to unravel further critical insights into VLCFA elongation in Arabidopsis.

## Data availability statement

The original contributions presented in the study are included in the article/**Supplementary Material.** Further inquiries can be directed to the corresponding author.

## Author contributions

JJ and FD conceived the project, FD, MB and JJ designed the experiments, MB, MA, SP, DT, RH, FB, FD and JJ performed the experiments, MB, FD and JJ wrote the article with contributions of all the authors. All authors contributed to the article and approved the submitted version. 
