# Supplementary material for: Tackling functional redundancy of Arabidopsis fatty acid elongase complexes
Source: Front Plant Sci. 2023 Jan 25;14:1107333. doi: 10.3389/fpls.2023.1107333 (PMC9928185; doi:10.3389/fpls.2023.1107333)
Supplement: Supplementary file 1 [file DataSheet_1.pdf]

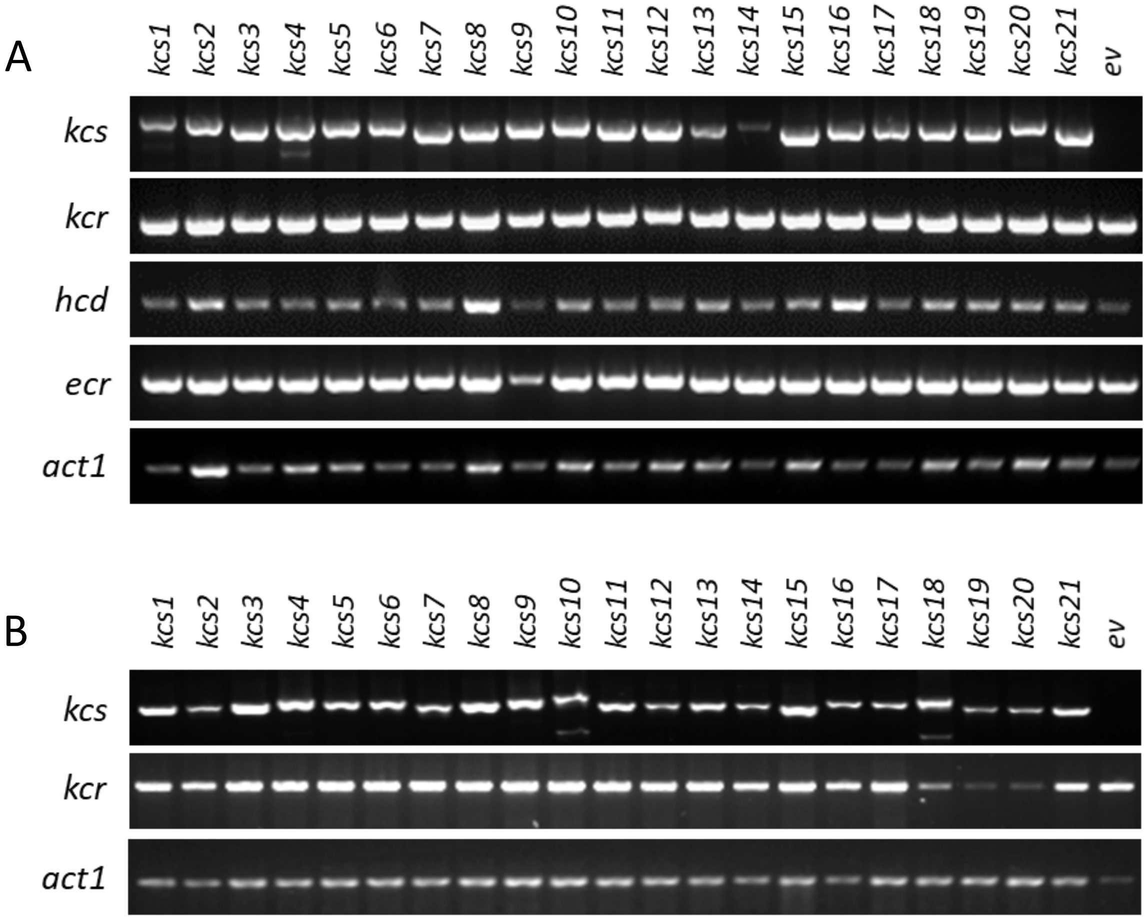

Supplemental Figure S1. RT-PCR analysis of steady-state KCS, KCR, HCD and ECR transcripts in INVSc1 TRIPLE (**A**) and INVSC1 TRIPLE Δ*elo3* (**B**) transformed with the different constructs as indicated. ev corresponds to the strain transformed with the empty vector. The yeast *actin1* (*act1*) gene was used as a constitutively expressed control.
