# Supplementary material for: Tackling functional redundancy of Arabidopsis fatty acid elongase complexes
Source: Front Plant Sci. 2023 Jan 25;14:1107333. doi: 10.3389/fpls.2023.1107333 (PMC9928185; doi:10.3389/fpls.2023.1107333)
Supplement: Supplementary file 2 [file DataSheet_2.pdf]

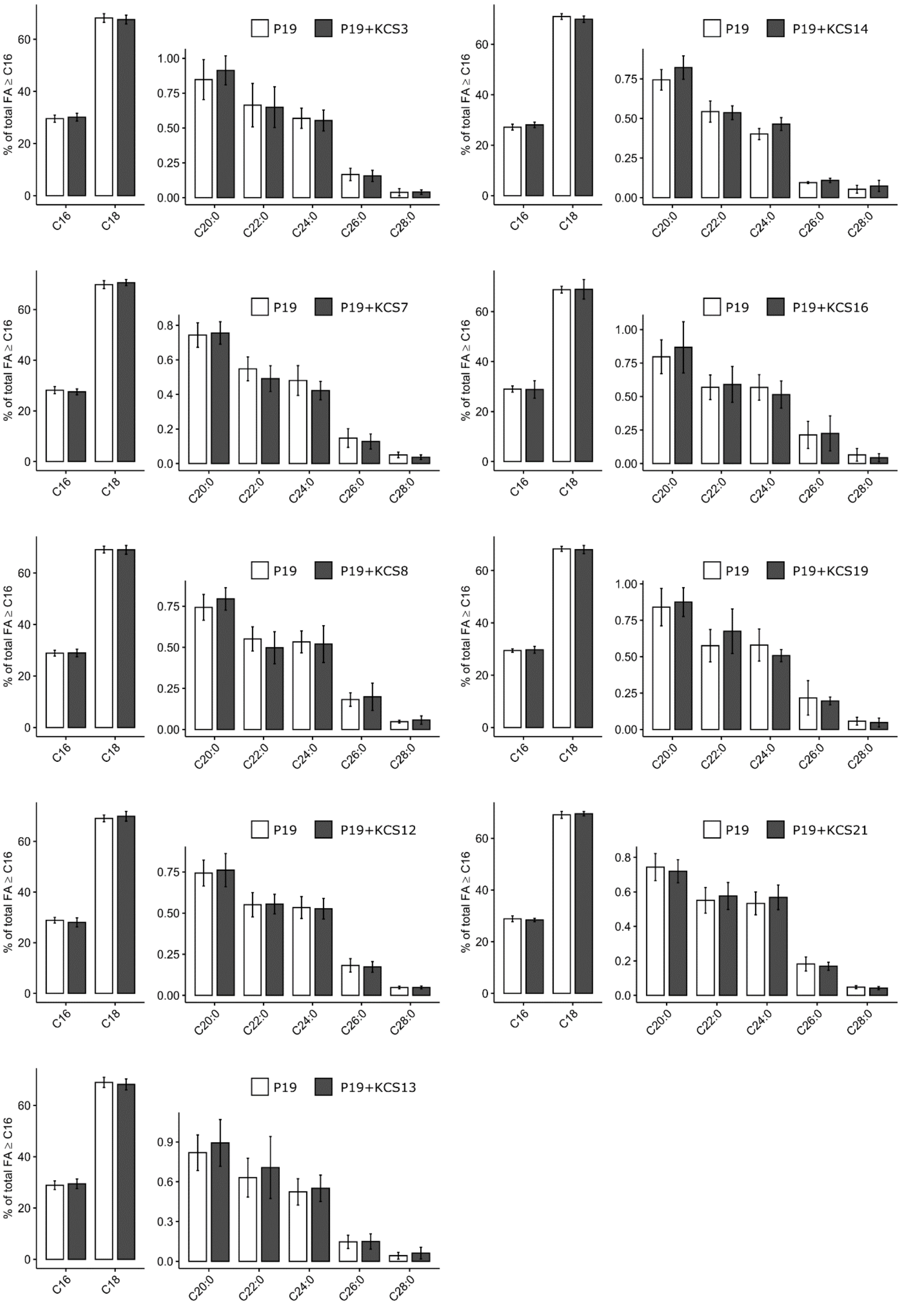

Supplemental Figure S2 : Heterologous expression of 9 AtKCS in *Nicotiana benthamiana* leaves. Comparison of FAMEs profiles obtained in leaves co-expressing KCS and the gene silencing suppressor P19 with the control condition (leaves transformed with P19 only). Mean values (percentage of total FA > or = C16) are given with SD (n>6). No significant changes were detected using Wilcoxon test.
